# Potentially inappropriate medications among elderly Brazilian outpatients

**DOI:** 10.1590/S1516-31802013000100004

**Published:** 2013-02-01

**Authors:** Christine Grützmann Faustino, Maria Cristina Guerra Passarelli, Wilson Jacob-Filho

**Affiliations:** I MSc. Pharmacist at the Faculdade de Medicina da Universidade de São Paulo (FMUSP), São Paulo, Brazil.; II PhD. Lecturer on Internal Medicine and Propaedeutics, Faculdade de Medicina do ABC (FMABC), São Paulo, Brazil.; III PhD. Titular Professor of Geriatrics, Department of Geriatric Medicine, Faculdade de Medicina da Universidade de São Paulo (FMUSP), São Paulo, Brazil.

**Keywords:** Geriatrics, Pharmacoepidemiology, Prescriptions, Ambulatory care, Aged, Geriatria, Farmacoepidemiologia, Prescrições, Assistência ambulatorial, Idoso

## Abstract

**CONTEXT AND OBJECTIVES:**

In Brazil, few studies have investigated the prevalence of potentially inappropriate medications (PIMs) among elderly outpatients. This study aimed to determine the prevalence of PIMs prescribed for elderly outpatients, identify the PIMs most commonly involved, and investigate whether age, sex and number of medications are related to prescription of such medications.

**DESIGN AND SETTING:**

Observational descriptive study developed in the Geriatrics Service of the Central Institute of Hospital das Clínicas (HC), Faculdade de Medicina da Universidade de São Paulo (FMUSP), São Paulo, Brazil.

**METHODS:**

Prescriptions issued to 1,270 elderly patients (≥ 60 years) were gathered from a database. These prescriptions had been written by geriatricians at a tertiary-level university hospital in São Paulo, Brazil, between February and May 2008. The prescriptions were divided according to sex and age group (60-69, 70-79 and ≥ 80). The Beers criteria were used to evaluate PIMs.

**RESULTS:**

Most of the sample comprised women (77%) and the mean age was 80.1 years. The mean prevalence of PIM prescriptions was 26.9%. Female sex and number of medications prescribed were associated with prescription of PIMs. The chance of having a PIM prescription was lower among patients ≥ 70 years.

**CONCLUSION:**

The greater prevalence of PIMs was correlated with female sex. The chance of having a PIM prescription was lower among patients ≥ 70 years and became greater with increasing numbers of medications prescribed (≥ 7).

## RESUMO

**CONTEXTO E OBJETIVOS:**

No Brasil, poucos estudos investigaram a prevalência de prescrição de medicamentos potencialmente inapropriados (MPIs) para idosos em ambulatório. Este estudo visa determinar a prevalência de MPIs prescritos para idosos no ambulatório, identificar os mais comumente envolvidos e verificar se a idade, o sexo e o número de medicamentos estão relacionados à prescrição de tais medicamentos.

**TIPO DE ESTUDO E LOCAL:**

Estudo descritivo observacional, desenvolvido no Serviço de Geriatria do Instituto Central do Hospital das Clínicas (HC) da Faculdade de Medicina da Universidade de São Paulo (FMUSP), São Paulo, Brasil.

**MÉTODO:**

Foram coletadas prescrições de 1.270 pacientes idosos (≥ 60 anos) em banco de dados. As prescrições foram realizadas por geriatras de hospital universitário de atenção terciária em São Paulo, Brasil, entre fevereiro e maio de 2008. As prescrições foram divididas de acordo com o sexo e faixa etária (60-69; 70-79 e ≥80). Os critérios de Beers foram utilizados para a avaliação de MPIs.

**RESULTADOS:**

A maior parte da casuística foi composta por mulheres (77%) e a média de idade foi de 80,1 anos. A prevalência média de prescrição de MPIs foi de 26,9%. O sexo feminino e o número de medicamentos prescritos foram associados à prescrição de MPIs. A chance de prescrição de um MPI foi menor em pacientes com ≥70 anos.

**CONCLUSÃO:**

A maior prevalência de MPIs encontrada foi correlacionada ao sexo feminino. A chance de prescrição de MPIs foi menor em pacientes com ≥ 70 anos e é maior à medida que aumenta o número de medicamentos prescritos (≥ 7).

## INTRODUCTION

Studies on aging as a factor relating to the response to medicine therapy gained prominence in the 1970s and 1980s. Investigations in this field began because of growing concern regarding the quantities of prescriptions (and their economic consequences), in parallel with demographic changes, and because it was a matter of consensus that elderly people were susceptible to unexpected effects from medicines.^1^

The presence of many comorbidities, high consumption of medicines, fragmented care, physiological changes and socioeconomic factors, among other factors, make it common for problems relating to medicines to arise among this population.^2^

This concern regarding the impact of prescriptions among an aging population has led to the creation of several strategies for dealing with this phenomenon. Among these is the detection of potentially inappropriate medicines (PIMs). Medicines are potentially inappropriate for elderly people if their indication is not based on evidence, if they increase the risk of adverse reactions in comparison with younger patients, or if they are not cost-effective. They are also associated with increased morbidity, mortality and expenditure of healthcare funds.^3^^,^^4^

Avoidance of the use of high-risk medicines is an important strategy for reducing occurrences of adverse effects from medicines, especially adverse reactions.^2^ Planning of interventions for promoting rational use of medicines requires knowledge of the prescriptions made for the elderly population.

## OBJECTIVE

The objectives of the present study were: to describe the prevalence of PIMs prescribed to elderly patients attended by the geriatrics service of a tertiary-level university hospital in São Paulo, Brazil, according to age group and sex, to identify the PIMs most commonly involved, and to investigate whether age, sex and number of medicines are related to prescription of PIMs.

## METHODS

An observational descriptive study was conducted on outpatient prescriptions written for patients attended at the Geriatrics Service of the Central Institute of Clinical Hospital, School of Medicine of the University of São Paulo (Instituto Central do Hospital das Clínicas da Faculdade de Medicina da Universidade de São Paulo, IC-HC-FMUSP) between February and May 2008. Data gathering was undertaken by means of generating reports from the database of the hospital administration and information system used by the outpatient pharmacy of HC-FMUSP. This system was designed and is maintained by the Data Processing Company of the State of São Paulo (Companhia de Processamento de Dados do Estado de São Paulo, Prodesp; a state-owned information technology company) and is used to dispense and control the stock of medicines. It has been in use since 2004 and is considered reliable for controlling these processes.

The prescriptions were divided according to sex and age group (60 to 69 years; 70 to 79 years; and ³ 80). Adults aged 60 years and over at the time of the data gathering were considered to be elderly, in accordance with the definition of the World Health Organization (WHO) for developing countries. Adults aged 80 years and over were considered to be very elderly. Age was calculated using the date of the prescription as the reference point.

Since this data gathering process took place over a four-month period, some patients appeared more than once on the spreadsheet, either with the same prescription or with different prescriptions. In these cases, only the first prescription for each patient was taken into consideration. The geriatric service is composed of different attendance subspecialties that are divided between general and specific types. Patients requiring special care (for example, those with advanced cognitive deficits or presenting obesity) are referred to subspecialties with specific attendance, while other patients are referred to subspecialties with general attendance. With the aim of diminishing the confounding factors in the analysis on the results, only the patients seen at subspecialties with general attendance were considered in this study.

Prescriptions for patients attended at outpatient services with brief consultations and prescriptions for patients who were not registered at the institution were not taken into consideration.

To evaluate the PIMs, the 2003 version of the Beers criteria were used, taking into consideration only the medicines that were not dependent on the diagnosis.^4^

The PIMs digoxin, ferrous sulfate and lorazepam were excluded from the analysis because it was not possible to calculate the dose. Although the drugs clonazepam and nitrazepam do not appear among the Beers criteria, they were considered to be PIMs because their half-lives are greater than 20 hours.^5^^,^^6^ Nitrazepam is on the market in Brazil, but not in the United States.^6^ The drug primidone is a barbituric anticonvulsant that does not appear among the Beers criteria but is routinely used in our institution. It is highly addictive and causes more adverse reactions among elderly people than do the majority of sedative or hypnotic drugs,^7^ and thus it was decided to consider this too as a PIM. The medicines were classified using the Anatomical Therapeutic Chemical (ATC) classification of the World Health Organization.^8^

For the statistical analysis, a logistic regression model was fitted to the data, taking male sex and the age group of 60-69 years as the reference points. The number of medicines was categorized according to the intervals defined by the following quartiles: 1 to 4; 5 to 6; 7 to 9; and ³ 10. The Hosmer-Lemeshow test was used to evaluate the fit of the models.^9^ For the hypothesis tests, the significance level was set at 0.05. The analysis was performed with the aid of the Minitab version 15 and Statistical Package for the Social Sciences (SPSS) version 11 applications.

## RESULTS

The analysis included 1,270 prescriptions. Most of the elderly patients were female (77.7%), and the number of women was greater than the number of men in all age groups. Among the women, the age group ^³^ 80 years formed the greatest proportion of the sample (Table 1). The mean age of all the patients attended was 80.1 years, while it was 80.2 years for the women and 79.9 years for the men.

**Table 1.** Frequencies and percentages of patients according to sex and age group, in relation to the total number of patients (São Paulo, 2008)

Age	Sex		Total
	Female	Male	
60-69 years	84 (6.6%)	21 (1.7%)	105 (8.3%)
70-79 years	367 (28.9%)	116 (9.1%)	483 (38%)
³ 80 years	536 (42.2%)	146 (11.5%)	682 (53.7%)
Total	987 (77.7%)	283 (22.3%)	1270 (100%)

The mean number of medicines prescribed was 7.5 (standard deviation, SD = 3.2), considering both sexes and all age groups. For the age group from 60 to 69 years, the mean was 7.5; for the age group from 70 to 79 years, it was 7.8; and for the age group of 80 years and over, it was 7.3. There were no statistical differences in the mean numbers of medicines per patient between the three age groups (P = 0.212), and this result did not depend on sex (P = 0.612). Taking all the age groups together, the mean number of medicines prescribed for the women was 7.8 (SD = 3.3) and, for the men, it was 6.5 (SD = 3.0). The mean number of medicines per patient was greater among the women (P < 0.001). This result did not depend on age group (P = 0.612).

The mean prevalence of PIM prescriptions was 26.9% (342 prescriptions). The age group from 60 to 69 years presented the highest prevalence (36.2%), followed by the age groups of 70 to 79 years (27.1%) and 80 years and over (25.4%). The group with the highest prevalence of PIMs was elderly women aged 60 to 69 years (Table 2).

**Table 2.** Frequencies and percentages of patients in the two categories of prescriptions of potentially inappropriate medicines (PIMs) (yes and no), according to sex and age group and according to Beers criteria 2003 (São Paulo, 2008)

Age	Sex	PIMs		Total
		No	Yes	
60-69 years	Female	53 (63.1%)	31 (36.9%)	84 (100%)
	Male	14 (66.7%)	7 (33.3%)	21 (100%)
70-79 years	Female	252 (68.7%)	115 (31.3%)	367 (100%)
	Male	100 (86.2%)	16 (13.8%)	116 (100%)
³ 80 years	Female	388 (72.4%)	148 (27.6%)	536 (100%)
	Male	121 (82.9%)	25 (17.1%)	146 (100%)
	Total	928 (73.1%)	342 (26.9%)	1270 (100%)

The mean number of PIMs per prescription was 1.17. With the exception of clonidine and dipyridamole, all of the PIMs involved are considered to have high severity of effects according to the Beers criteria, 2003 version (Table 3). Nitrazepam was not prescribed for any of these patients.

**Table 3.** Potentially inappropriate medications (PIMs) that were most prescribed, according to sex and according to Beers criteria 2003 (São Paulo, 2008)

PIMs	Sex	
	Female	Male
HC muscle relaxant^*^	204 (59.3%)	23 (42%)
Fluoxetine	38 (11%)	1 (1.8%)
Amitriptyline	23 (6.7%)	1 (1.8%)
Clonidine	16 (4.7%)	5 (9%)
Amiodarone	11 (3.2%)	4 (7.2%)
Bisacodyl	11 (3.2%)	5 (9%)
Clonazepam	9 (2.6%)	4 (7.3%)
Hydroxyzine	9 (2.6%)	1 (1.8%)
Mineral oil	7 (2%)	3 (5.5%)
Oxybutynin	5 (1.5%)	1 (1.8%)
Primidone	4 (1.1%)	3 (5.5%)
Methyldopa	3 (0.9%)	---
Nitrofurantoin	2 (0.6%)	3 (5.5%)
Chlorphenamine	1 (0.3%)	---
Naproxen	1 (0.3%)	---
Dipyridamole	---	1 (1.8%)
Total	344 (100%)	55 (100%)

^*^Produced by the pharmacotechnical unit of the Pharmacy Division of the Central Institute of the Clinical Hospital, School of Medicine of the University of São Paulo (Instituto Central do Hospital das Clínicas da Faculdade de Medicina da Universidade de São Paulo, IC-HC-FMUSP). Contains carisoprodol 100 mg (PIM), dipyrone 200 mg and paracetamol 200 mg.

Female sex and prescription of ³ 7 medications were associated with prescription of PIMs (Table 4), but this was not observed in relation to the age group ³ 70 years. The Hosmer-Lemeshow test showed that the model was a good fit (P = 0.703).

**Table 4.** Factors associated with prescription of potentially inappropriate medications, according to Beers criteria 2003 (São Paulo, 2008)

Characteristic	P value	Odds ratio	95% confidence interval
Female sex	0.018	1.5	1.1-2.2
Age			
70-79 years	0.031	0.59	0.37-0.91
³ 80	0.024	0.59	0.37-0.91
Medications			
1 to 4	0.065	----	----
5 to 6	0.065	----	----
7 to 9	< 0.001	4.5	2.7-7.5
³ 10	< 0.001	9.2	5.5-15.3

## DISCUSSION

The prevalence of PIMs in this study (26.9%) was within the range found in studies in other countries in which the investigators used the 2003 version of the Beers criteria to evaluate the prescriptions (13 to 40.7%).^2^^,^^10^^,^^11^^,^^12^^,^^13^ Buck et al. studied the databases of two American hospitals and found that the prevalence of PIMs among elderly outpatients was around 23% in both hospitals.^14^ In a study on 50 medical files from elderly patients who were attended at a geriatric service, Maio et al. observed that the prevalence of PIMs was 26%.^15^ Like in the present study, the instruments chosen by the investigators in other studies were adapted according to the availability of the data gathered and the list of medications available in the institution or approved in each country.

In Brazil, Carvalho found that the prevalence of PIMs was around 15.4%, in a population sample from the year 2000, using the 2003 version of the Beers criteria.^16^ Mosegui et al.^17^ observed that 17% of the medications were inappropriate for the indications for which they had been prescribed, and this was close to the proportion found by Coelho Filho et al. (20%).^18^ Also using the 2003 version of the Beers criteria, Gorzoni et al. observed that 41% of the elderly people evaluated were making use of one or two PIMs.^19^ Almeida et al. found that the prevalence of PIMs was 18.5% among elderly people attended at a mental health service in São Paulo, using the modified Stuck criteria.^20^

If on the one hand, we found percentages that were close to those in the literature, on the other hand it was difficult to compare the results. The prevalences in different populations vary according to the time and place of data gathering, and according to other factors such as the criteria used. Furthermore, the study design and duration of data gathering may also contribute towards the differences.^21^

The chance of prescribing a PIM was smaller among patients ³ 70 years, and this tendency has also been observed by other authors.^21^^,^^22^^,^^23^^,^^24^ Piecoro et al. found that elderly Americans ³ 85 years of age were less likely to receive PIMs^21^ while Passarelli et al. found that the use of PIMs was significantly lower among elderly patients ³ 80 years of age who were admitted to a Brazilian teaching hospital.^24^ However, there is no consensus in the literature regarding whether prescribing of PIMs increases or decreases as patients grow older.^25^^,^^26^^,^^27^ Stuck et al. observed that patients ³ 80 years of age had a greater tendency to use PIMs^25^ and Lechevallier-Michel et al. found that the frequency of use of PIMs increased with age.^26^

In our study, the women presented greater likelihood of having PIMs prescribed than did the men. This association has also been reported in other studies,^21^^,^^22^^,^^26^^,^^28^ including in the only Brazilian study that set out to investigate this relationship.^18^ However, Gallagher et al. and Pitkala et al. did not find any statistical difference between the sexes in relation to PIM prescription.^27^^,^^29^ We do not know why women are more exposed to PIM prescription, but we observed in our study that they presented a higher mean number of medications prescribed than did the men (7.8 versus 6.5), which may have influenced this association. Research is needed to elucidate the dynamics of differences between the sexes, in interactions between providers and patients and in experiences within healthcare systems, that expose women to receiving greater numbers of medications.^28^ For example, if women tend to report pain and depressive symptoms more than men do, there is a greater likelihood that these women will be diagnosed and treated under these conditions. On the other hand, men may be less exposed to these medications because they may not have reported the symptoms.^28^ It is likely that women show greater concern regarding their health than men do, which would imply a higher mean number of items prescribed.^28^

It was observed in this study that the chance of PIM prescription increased as the number of items prescribed increased: the odds ratio was 4.5 times greater if the prescription contained 7 to 9 medications and 9 times greater if it contained 10 or more. This tendency was also in agreement with what has been found in other studies.^14^^,^^21^^,^^23^^,^^26^

The PIMs that were most prescribed for the women were carisoprodol, fluoxetine and amitriptyline, while for the men, they were carisoprodol, clonidine and clonazepam. For both the men and the women, carisoprodol was the PIM that was most prescribed.

The adverse reactions from carisoprodol include lethargy, agitation, delirium, psychosis and hepatic toxicity.^30^ The presentation of this drug that is made available at our institution has other disadvantages for elderly patients: there are no clinical studies in the literature that we consulted that confirm the efficacy of using this in associations for elderly people; when this medication is prescribed, patients will receive three different types of drugs, which increases the polypharmacy and presents risks to patients who are allergic to any of the components; and the adverse reactions from the drugs may be mutually exacerbated, such as hypotension and liver failure.^30^ Furthermore, analgesics were one of the drug subclasses most prescribed for both the men and the women. In the cases in which HC (carisoprodol 100 mg, dipyrone 200 mg and paracetamol 200 mg) muscle relaxant was also prescribed, overlapping between medications with the same therapeutic purpose may be occurring. In Brazil, there are no controls over the sale of carisoprodol.

In therapeutic classes in which few options exist, such as muscle relaxants or gastrointestinal antispasmodic drugs, it is much more difficult for prescribers to change the therapy. Since all muscle relaxants may cause sedation, elderly people for whom this class is prescribed need to be carefully monitored in order to prevent adverse effects, if the medication cannot be changed.^31^ Gray and Gardner cited an example in which an institution dealt with this question.^32^ Educative presentations to physicians have included suggestions for safer medications, along with non-pharmacological interventions for treating muscle pain.^32^

In other studies that used the 2003 version of the Beers criteria, the PIMs that were most prescribed were long-action benzodiazepines, propoxyphene, amitriptyline and anti-histamines.^10^^,^^12^^,^^14^^,^^27^^,^^33^ Estrogen, muscle relaxants, ticlopidine, chlordiazepoxide and anti-inflammatory agents have also been cited.^12^^,^^27^^,^^33^ In Brazil, Carvalho observed that anti-inflammatory agents, methyldopa, digoxin and benzodiazepines with long half-life were the PIMs most used by elderly people in the city of São Paulo.^16^ Gorzoni observed that benzodiazepines, methyldopa, ergot derivatives and cyclandelate were the drugs most often found in analyses on medical files.^19^ Passarelli et al. found that the PIMs most commonly encountered among elderly people who were admitted into wards were diazepam, amiodarone, nifedipine, methyldopa and cimetidine.^24^

Differences in the profiles of PIM prescriptions may have occurred for several reasons. Some of the medications within the Beers criteria are not routinely used in our institution, such as chlorpropamide, guanethidine, reserpine and cimetidine. Others have not been registered with the National Sanitary Surveillance Agency, such as oxazepam, quazepam, halazepam and doxepin, and these are therefore not on the market in Brazil.

Our service does not have interdisciplinary activities that could influence prescriptions, such as reviews conducted by pharmacists. Such interventions have been shown to be effective for optimizing drug therapy among elderly patients in other countries.^34^

The mean number of medications prescribed for the elderly people in our study (7.5) was close to the numbers found by Bierman et al. and Carey et al. In an investigation using an American database on 965,756 patients aged 65 years and over who were attended at hospitals within the Veterans Health Administration between 1999 and 2000, Bierman et al. found that the mean number of items received was 7.2 per patient.^28^ In an analysis on records relating to 230,00 patients in a database in the United Kingdom between 1996 and 2005, Carey et al. observed that the mean number of items prescribed tended to increase over the years, and it was 7.4 in 2005. In their study, the data considered included not only outpatients but also home care patients.^35^ Nonetheless, most studies in other countries have presented lower mean numbers of medications prescribed for elderly outpatients, ranging from 3.7 to 6.8 items.^14^^,^^27^^,^^36^^,^^37^

The mean consumption among elderly Brazilians who used medications (prescribed and non-prescribed) has also been found to be lower, ranging from 1.3 to 4.3 per elderly person in the studies carried out.^16^^,^^17^^,^^18^^,^^19^^,^^38^^,^^39^ The main investigation methods used have been population-based surveys or household interviews. Although the Brazilian pharmaceutical market is one of the largest in the world (in 2008, the national pharmaceutical industry made sales of more than R$ 30 billion),^40^ there are no databases appropriate for wide-ranging pharmacoepidemiological investigations on drug prescription or consumption, unlike in other countries.^41^

In the present study, the age group from 70 to 79 years presented the highest mean number of medications prescribed. After this peak, there was a tendency for prescription to diminish, which makes us suppose that increasing care was taken regarding drug therapy among very elderly patients. Professionals writing prescriptions for patients within this age group probably pay much closer attention to them, because of their physiological changes and associated comorbidities, and the limitations that they present.

The tendency towards higher mean numbers of medications prescribed for women that was observed in the present study is in line with what has been seen in studies in other countries^42^^,^^43^^,^^44^ and in other Brazilian studies.^18^^,^^45^

If, on the one hand, polypharmacy causes concern because it has been correlated with adverse reactions and drug interactions,^46^ on the other hand excessive concern regarding the negative aspects of polypharmacy and attempts to avoid prescribing many medications may give rise to a situation in which less attention is paid to the fact that all medications that patients need must be tried out.^47^^,^^48^ It can be seen that the boundary between rational and empirical use of drugs among elderly people is narrow. Decision-making is often impaired through lack of evidence or, at best, evidence that is only minimally adequate for this population.^49^

The Beers criteria present limitations. The list of PIMs in this instrument has the disadvantage of being inflexible, without taking differences between individuals into account, which may lead to false positive signs (for example, indicating that there is a problem that in reality does not exist). Furthermore, the criteria do not show up problems that have not been described and thus they will fail to provide a complete evaluation on patients.^50^^,^^51^

Some medications are not completely contraindicated for elderly people, especially among patients with short life expectancy, for example amitriptyline, bisacodyl and naproxen.^52^ The criteria do not mention underuse of medications or drug-to-drug interactions, and they do not make reference to duplicated therapeutic classes. The presentation of medications in the list is confusing, since they are not listed according to alphabetical classification, sites of action or therapeutic classification.^53^

Among the positive aspects of using the Beers list are the facts that it can be adapted to computer systems, it enables pharmacoepidemiological studies on large populations, it incorporates information from the specialized literature and from specialists’ consensuses, and it can easily be used for educational purposes.^2^^,^^49^

To obtain the results from this study, a spreadsheet generated through a database was used. The advantages of using a computerized database for investigating prescribed medications include the accuracy of registering what was prescribed and the fact that it does not depend on information supplied by patients. The prescription sample that we used was gathered over a considerable period (four months), and care was taken to ensure that only the subspecialties with general attendance were taken into consideration in evaluating the prescriptions. On the other hand, adaptation of a spreadsheet that was designed for administrative use, for it to be reused for research, made it necessary to draw up complex formulas and standardize the alphanumeric data. Such stages made it difficult for the institution’s healthcare professionals to conduct pharmacoepidemiological studies.

The present study presents certain limitations. Because of the many comorbidities involved, the elderly people in the sample may also have been attended by other specialized services, which could have influenced the prescription profile. Failure to diagnose certain conditions, or misdiagnosis, may also have influenced the prescriptions. There may have been typing errors in the prescriptions, although the triple-checking of the typing at the outpatient pharmacy suggests that such errors had little influence on the data obtained.

The system that generated the spreadsheet used in this study did not have any interface with electronic medical record systems. Thus, the diagnoses and other information that formed the basis for the physicians’ prescriptions were not available. Because of this, it was not possible to determine whether the drugs that were considered to be PIMs in this study were actually potentially inappropriate for certain patients. Moreover, without monitoring the treatment outcomes, the Beers criteria do not allow it to be confirmed that adverse reactions actually occurred, or what form they took. These criteria only indicate that there would be a greater likelihood of occurrences of adverse reactions in elderly individuals.^21^

Only limited generalization of the results to the general population can be made, because this study was restricted to the prescription profile of patients attended at a tertiary-level healthcare institution. In many cases, these patients had been referred to these services because of the complexity of their comorbidities.

Some authors have suggested that, to deal with the complexity of drug treatment among elderly people, it is necessary to select patient profiles, comorbidities, therapeutic classes or drug classes that are more closely related to occurrences of negative outcomes, with the aim of prioritizing groups that are at risk regarding the appearance of drug-related problems. For other authors, the use of medications among elderly people is a wide-ranging question that requires an interdisciplinary approach. For this to happen, it is of fundamental importance to have constant improvements in information recording and access relating to the use of medications. The prescription profile that is determined should be easily accessible to the team of healthcare professionals. Electronic medical record systems in which the records are adequately completed, with connections to the prescription database, would make it possible to carry out systematic in-depth evaluations on different aspects of the use of medications.^54^

Secondly, the professionals dealing with elderly patients need to know what the appropriate prescription practices are. This can be achieved through access to protocols for drug use and actions relating to continuing education. Knowledge of appropriate practices would diminish the chances of occurrences of potentially inappropriate practices.

Lastly, it is of fundamental importance to emphasize that there is a need to create a list of PIMs that is adapted to Brazilian realities. Such a list, obtained through studies on drug use, consensuses and evidence-based literature, could guide the selection of medications and preparation of protocols for drug use among elderly people.^54^

## CONCLUSION

The mean prevalence of PIM prescriptions was 26.9% (342 prescriptions). The age group from 60 to 69 years presented the highest prevalence (36.2%), followed by the age groups of 70 to 79 years (27.1%) and 80 years and over (25.4%). With the exception of clonidine and dipyridamole, all of the PIMs involved are considered to have high severity of effects according to the Beers criteria, 2003 version. Female sex and prescription of ³ 7 medications were associated with prescription of PIMs, but this was not observed in relation to the age group ³ 70 years. The prevalence of PIMs in this study was within the range found in studies in other countries in which the investigators used the 2003 version of the Beers criteria to evaluate the prescriptions, but only limited generalization of the results to the general population can be made, because this study was restricted to the prescription profile of patients attended at a tertiary-level healthcare institution.
